# Heating Characteristics and Induced Healing Efficiencies of Asphalt Mixture via Induction and Microwave Heating

**DOI:** 10.3390/ma11060913

**Published:** 2018-05-29

**Authors:** Quantao Liu, Cheng Chen, Bin Li, Yihan Sun, Hechuan Li

**Affiliations:** 1 State Key Laboratory of Silicate Materials for Architectures, Wuhan University of Technology, Luoshi Road 122, Wuhan 430070, China; liuqt@whut.edu.cn (Q.L.); chencc@whut.edu.cn (C.C.); lib@whut.edu.cn (B.L.); 2 National & Local Joint Engineering Laboratory for Transportation and Civil Engineering Materials, Chongqing Jiaotong University, Chongqing 400074, China; 3 Zhejiang Provincial Institute of Communications Planning, Design & Research, Hangzhou 310015, China; sunyh@whut.edu.cn

**Keywords:** asphalt mixture, microwave heating, induction heating, effective heating depth, induced healing

## Abstract

This paper investigates the heating characteristics and induced healing efficiencies of asphalt mixture containing steel fiber under induction heating and microwave heating. The heating characteristics of an asphalt mixture with different heating methods were studied with an infrared camera. The healing performance of the asphalt mixture specimens in different healing conditions were investigated by observing the crack closure and testing the fracture resistance recovery after healing. The results showed that the heating speed at the surface of asphalt mixture with induction heating was much higher than that with microwave machine heating, under a similar output power and the same method of radiation. While the temperature distribution within the asphalt mixture under induction heating was quite uneven, microwave heating resulted in a more uniform temperature distribution. The effective heating depth of microwave heating is much higher than that of induction heating. Gradient healing occurred within the sample heated with induction healing, while a uniform healing effect can be achieved with microwave heating.

## 1. Introduction

Asphalt is a typical temperature-sensitive material, and its self-healing ability is highly dependent on temperature. Heating has been used by many researchers to enhance the self-healing properties of asphalt and asphalt mixtures [1,2,3,4,5]. Electromagnetic induction, microwave, and infrared heating methods were widely applied to increase the temperature of an asphalt mixture for healing purpose [6,7]. A suitable temperature can lead to low viscosity and good flow performance of asphalt binders, resulting in significant improvement of the self-healing properties of the asphalt binder and the restoration of micro-structural damages within the asphalt mixture [8,9].

It has been demonstrated that induction heating can be used to improve the healing capability of asphalt mixtures [10,11,12]. During induction heating, asphalt mortar containing conductive particles is exposed to an alternating electromagnetic field with a frequency in the order of kilohertz [13]. Induction energy heats the metallic fibers by means of high-frequency alternating electromagnetic fields, which are able to induce eddy currents in materials that are electrically and magnetically susceptible. The heat energy diffuses into the asphalt binder to increase the temperature. Asphalt mixture can be healed quickly, because asphalt binder behaves as a near-Newtonian fluid when its temperature is above the softening point of the binder [14]. Liu et al. prepared electrically porous asphalt mixture by adding steel fibers, and proved that this kind of asphalt concrete can be easily heated via induction heating [15]. It was also demonstrated that long steel wool with a smaller diameter is more effective than short steel fiber with a bigger diameter for increasing the temperature [16]. Wang et al. and Dai et al. also studied the induced healing capability of asphalt mastic and dense-graded asphalt mixture beam samples through induction heating technology [17,18]. It was found that the micro-cracks in the mastic samples could be effectively healed at a heating temperature of 85 °C, whilst 100 °C was identified as the maximum allowable induction heating temperature for asphalt mixtures [15]. 

Microwave heating is also considered as a promising technique to promote the self-healing of composite materials with metallic fibers [19,20]. Microwave heating causes the polar molecules’ orientation to change due to the alternating magnetic field. As a result, the movement of the molecules is disturbed and hindered, and the temperature rises. In addition, if ferrous particles are added to the mixture, they may reflect microwave radiation and accelerate the increase of temperature [21,22]. Ferrous particles can be used to increase heating rates of asphalt mixtures, because they can absorb and conduct more thermal energy than the other components of the mixtures, such as aggregates and asphalt binder. Laboratory experiments have been conducted by adding steel fibers into an asphalt mixture induce healing of that asphalt mixture with microwave heating. Al-Ohaly et al. studied the effects of microwave heating on asphalt adhesion as well as water damage in asphalt mixtures. They emphasized that microwave treatment had the potential to improve the bonding properties of asphalt and aggregate [23]. Sun et al. studied the induced-healing performance of asphalt mixture under microwave irradiation, and proved that microwave heating induced a good heating effect for asphalt mixture containing steel slag [24,25]. 

It is widely accepted that heating can effectively enhance the induced healing ratios of asphalt mixtures. However, there is debate over which heating method is superior. To compare the heating characteristics and crack healing performance of asphalt mixture via induction heating and microwave heating, and find out which heating technology is more superior, this paper investigates the heating rate, heating uniformity, effective heating depth, and healing ratio of asphalt mixture induced by these two heating methods.

## 2. Materials and Experiments

### 2.1. Materials 

Penetration-grade 70# asphalt binder obtained from Hubei Guochuang Hi-tech Material Co. Ltd. of Wuhan, China was used in this paper. Steel wool fibers provided by Shanghai Auticar Metal Products Co. Ltd. (Shanghai, China) were used as the heating units for asphalt mixtures with induction and microwave heating. The properties of the asphalt binder and steel wool fibers were shown in Table 1, where Chinese Technical Specification JTG F40-2004 was used. The optimal content of steel wool fibers was 6% by volume of the asphalt binder, according to previous research [26]. Asphalt mixture with 6% steel wool fibers has the highest strength and particle loss resistance, and an acceptable induction heating speed. Basalt aggregates and limestone filler were used as the mineral materials in this research.

### 2.2. Sample Preparation

AC-13 asphalt mixture was designed in this paper according to the Marshall Design method, and the asphalt–aggregate ratio was 5.0%. The mixture was mixed and compacted at 160 °C and 150 °C respectively. The air voids (VV) were 4.4%, voids in mineral aggregates (VMA) were 14.3%, and voids filled with asphalt (VFA) were 69.4%. The volume indices of asphalt mixture were tested according to Chinese standards; all the indices met the standard requirements [26]. Asphalt mixture rutting slabs were formed with the wheel-rolling compaction method, according to Technical Specification JTG E20-2011, and divided into several beams with a length of 200 mm, width of 40 mm, and height of 40 mm. A notch with a depth of 5 mm and a width of 4 mm was sawn into the center of the beams to control the position of failure during the fracture test. Asphalt slabs with a length of 30 cm, a width of 30 cm, and a height of 10 cm were also prepared; cores with diameter of 10 cm were drilled in the middle of slabs. The slabs were used for heating under induction and microwave machines, and the cores were used to analyze the vertical temperature distribution after heating with the two heating methods. 

### 2.3. Induction and Microwave Heating

**Figure 1:**
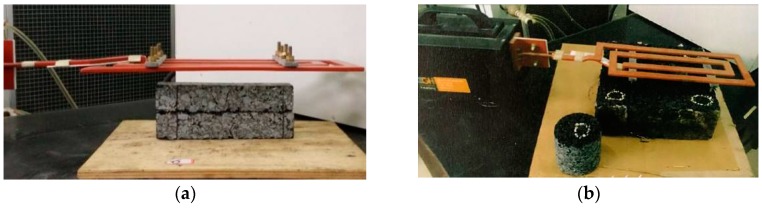
Induction heating of the sample: (**a**) hearing beam, (**b**) heating slab.

As shown in Figure 1, asphalt samples were heated by an induction heating machine with an output power of 8.3 kW and a frequency of 123 kHz. The distance between the coil and the surface of sample has a significant influence on the heating speed. The distance used in this paper for heating beams was 10 mm to obtain a high heating speed, according to the preliminary study [27], and 30 mm for slabs, the same distance as the microwave machine on slabs.

**Figure 2:**
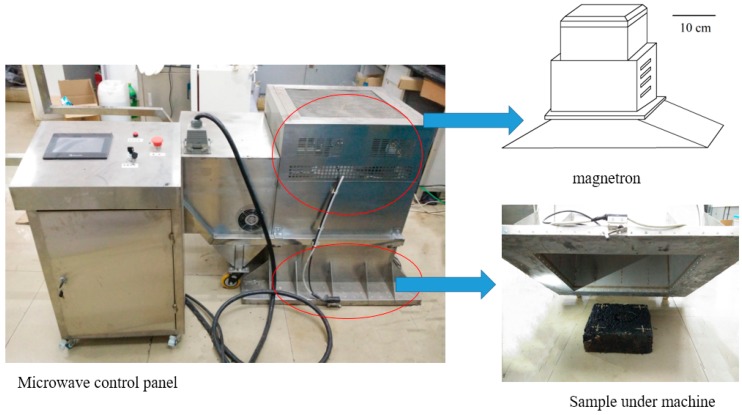
Improved microwave heating machine for slabs.

As shown in Figure 2, a self-developed microwave machine with a frequency of 2.45 GHz and an output power of 5 kW was also used in this research. This improved microwave machine contains a microwave magnetron, control panel, and metal cover (to prevent microwave leak). In order to simulate the practical microwave heating process of asphalt pavement, the microwave launcher was installed in the upper section of the metal cover, and the distance between the launcher and asphalt mixture slabs was 30 cm. The microwave launcher radiates waves from the top of the machine, and directly irradiates on the top surface of the samples being heated. This way, the microwave heating can be comparable with induction heating. An infrared image camera with a resolution of 320 × 240 pixels was used to determine the temperature distribution within the samples after induction and microwave heating. 

### 2.4. Fracture and Healing Test

**Figure 3:**
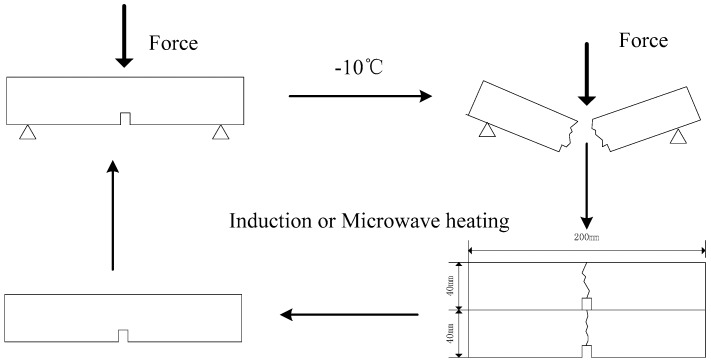
Healing test procedure of beam samples.

A universal testing machine (UTM-25, IPC Global, Victoria, Australia) was used to conduct the fracture and healing test. The schematic view of the beam samples and the fracture-healing procedure are shown in Figure 3. 

All samples were kept in the freezer for 6 h at −10 °C before the fracture test to decrease the viscoelasticity and unrecovered deformation of the samples during the fracture test. The asphalt beams were broken under a three-point bending setup, to obtain the original fracture force F_1_. The distance between the two supporting points was 160 mm. Loads were applied on the samples with a descending speed of 50 mm/min; the force-displacement curves of the samples were recorded by a computer connected with the loading machine. The fractured beams were placed on a plate until they reached room temperature, and two broken beams were stacked together for heating treatment with induction heating or microwave heating. After the heating process, the samples were cooled to room temperature and fractured again at −10 °C, as in the first fracture test. The second fracture force F_2_ was obtained, and the healing ratio induced by induction; microwave heating was defined as the maximum force after healing divided by original fracture force. 

## 3. Results and Discussion

### 3.1. Heating Characteristics of Asphalt Mixture with Induction Heating

**Figure 4:**
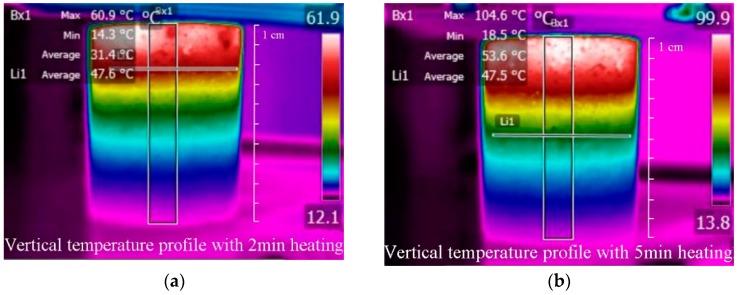
Infrared images of the core after induction heating at a heating distance of 30 mm. (**a**) Vertical temperature profile of the sample with 2 min heating. (**b**) Vertical temperature profile of the sample with 5 min heating.

The sample showed a high heating efficiency with induction heating technology. The infrared images of the sample after 2 and 5 min heating were shown in Figure 4. It can be seen that the sample showed a very obvious rainbow temperature distribution, ranging from 14.3 to 60.9 °C after 2 min heating, which means a gradient heating in the vertical direction. The heating speed decreased gradually with increasing depth. The top part of the core shows an obvious heating performance, thus the crack in this layer can probably healed soon. The temperature at the bottom of the core was 14.3 °C after 2 min heating, which is not sufficient to induce the healing of the binder. Therefore, an effective healing depth exists for the sample being induction-heated and it is less than the thickness of the sample. 

The healing efficiency of an asphalt mixture depends on the capillary flow speed of the binder. The softening point of asphalt binder can be considered as a critical healing temperature for the binder to obtain a good healing property [14]. The softening temperature of the 70# base bitumen used in this paper was 47.5 °C. So the depth at which the temperature reached 47.5 °C was considered as effective healing depth of the sample. At the depth of 2.2 cm, the temperature was 47.6 °C (around the softening point of the binder). Therefore, 2.2 cm was considered as the effective heating depth with 2 min of induction heating. After 5 min of heating, the average temperature at the surface of the slab was 99.8 °C (the heating rate at the surface of the slab was 17.5 °C/min), the allowable surface temperature. As shown in previous research, the surface heating temperature should not be higher than 100 °C, to avoid the swelling of the binder and the excess expansion of the mixture [28]. Therefore, no further heating can be applied, to avoid overheating of the sample surface. In this case, the temperature was 47.6 °C (around the softening point of the binder) at the depth of 4.8 cm. Therefore, 4.8 cm was considered as the maximum heating depth with induction heating. Even within the effective healing depth of 4.8 cm, both the temperature and the induced-healing ratio are not uniform. As temperature is the key parameter controlling the healing rate of the mixture, the upper part of the sample will be healed better than the lower part.

**Figure 5:**
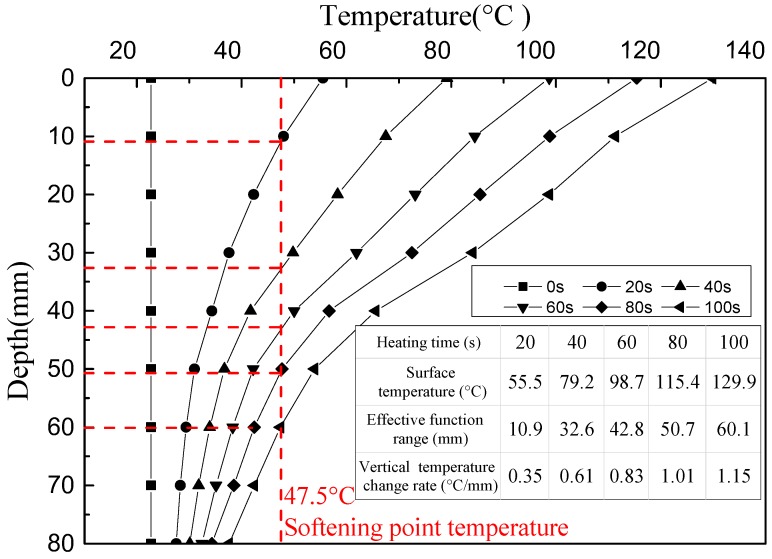
Vertical temperature distribution of the samples at a heating distance of 10 mm.

The heating distance between the coil and the top surface of the sample can significantly influence the effective heating depth of an asphalt mixture. A heating distance of 10 mm was recommended for the practical application of induction heating on asphalt pavement [15]. The vertical temperature distribution and the effective heating depths of the asphalt concrete samples with different heating periods, at a heating distance of 10 mm, are shown in Figure 5. It can be seen that the effective healing depth increased with the increase of the heating time. The effective healing depth was 10.9 mm after heating for 20 s, while the depth increased to 60.1 mm after 100 s of heating. However, the surface temperature of the sample reached 129.9 °C after heating for 100 s, and the beam sample suffered serious swelling deformation. At 60 s of heating, the sample surface approached its allowable heating temperature of 100 °C, and the effective depth of asphalt mixture with induction heating was around 42.8 mm, which can be assumed to be the maximum effective depth of asphalt mixture at this heating distance. Extending the heating time further will result in excess expansion deformation of the mixture. It can be noted that a smaller heating distance between the coil and the sample (higher heating speed) resulted in a lower effective heating depth of the mixture.

### 3.2. Healing Performance of an Asphalt Mixture at Different Induction Heating Temperature Gradients

**Figure 6:**
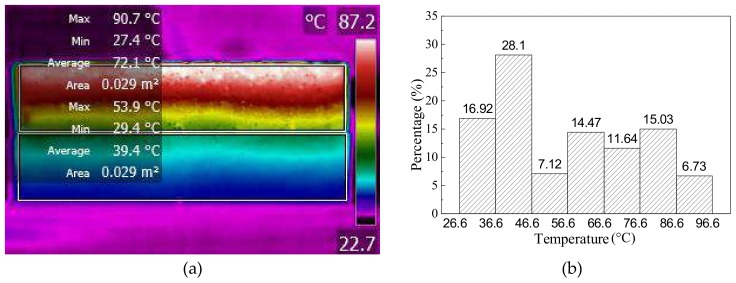
The (**a**) infrared image and (**b**) temperature distribution of the beam sample with 60 s induction heating.

Figure 6a shows the infrared image of beams after 60 s induction heating at a heating distance of 10 mm. It can be seen from the infrared image that sample showed a good heating effect, especially at the top layers. The temperature distribution presented a rainbow-like gradient, which shows an uneven heating effect in the vertical direction. It can also be seen that the average temperatures of upper and lower beams were 72.1 °C and 39.4 °C, respectively, and the temperature difference between upper and lower beams reached 32.7 °C. The temperature distribution of the beams was analyzed from the infrared image shown in Figure 6b. It is worth noting that the ratio above 46.6 °C (around softening point of asphalt) was 55%, meaning that half of the cracks can be healed at this critical healing temperature, according to Tang’s results [14].

In order to obtain the healing efficiency at different depths of the sample, two asphalt beams were stacked together during heating and separated after cooling. The damaged area was photographed in the same position before and after healing, to observe the healing phenomenon of the cracks. The maximum fracture forces before and after healing were measured, and the healing ratio of upper and lower beams was obtained. The healing behaviors of the mixture at three temperature gradients (60 °C, 80 °C, 100 °C, as well as surface temperature) were investigated. 

**Figure 7:**
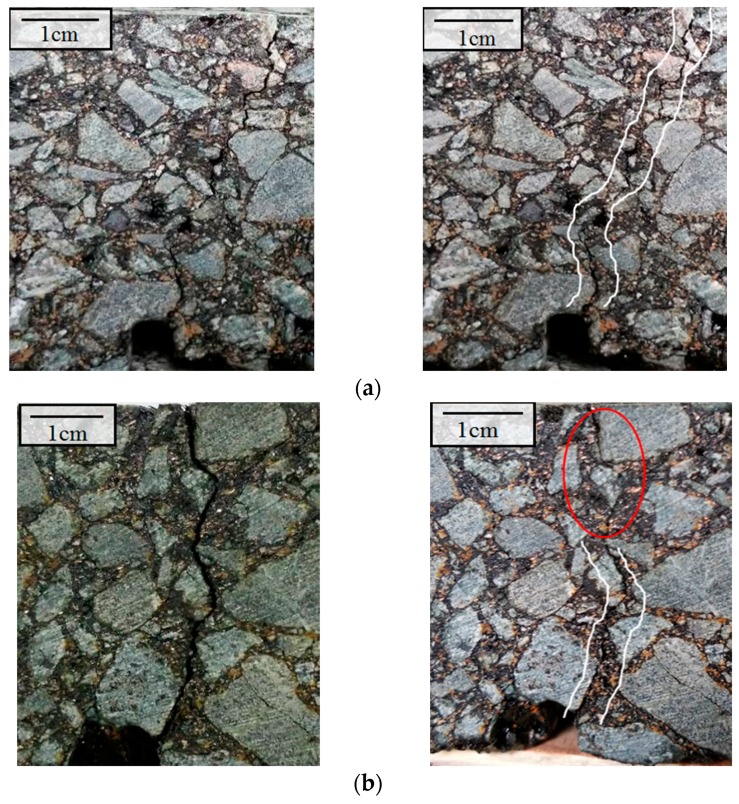

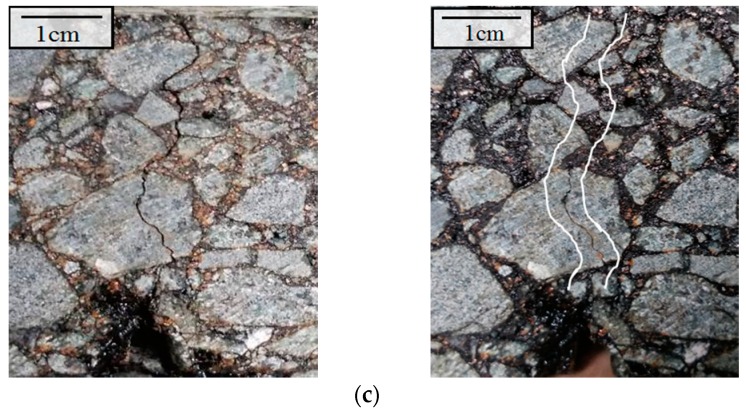
Healing phenomenon at different temperatures under induction heating. (**a**) Crack healing phenomenon at 60 °C: crack not healed; (**b**) Crack healing phenomenon at 80 °C: crack partially healed; (**c**) Crack healing phenomenon at 100 °C: fully healed, except for broken stone.

The crack healing phenomenon of the upper beam at different temperatures under induction heating is shown in Figure 7. The gradient healing phenomenon of the beam can be seen by comparing the cracks before and after healing. When the surface temperature of the sample was 60 °C, the crack was not healed, and can be seen clearly after heating due to low heating temperature and the resultant limited healing effect. As shown in Figure 7b, the shallow crack disappeared when the sample surface was heated to 80 °C, while the crack in the lower part can still be seen clearly after healing. This means that the healing effect of the sample tends to deteriorate with as the depth increases. When the surface temperature of the beam reached 100 °C, all cracks disappeared, but partial deformation can be seen at the surface of beams. It should be noted that some broken aggregates cannot be healed, because induced healing only occurs within asphalt mastic due to asphalt flow. The broken of the aggregates will significantly affect the strength recovery ratio of the samples.

Table 2 shows the induction healing ratios of the samples with different surface heating temperatures. It can be seen from the healing efficiency results that the healing ratios of the upper beams increased with the increase of the surface temperature. The healing ratio of the sample at surface temperatures of 60 °C, 80 °C, and 100 °C was 30.8%, 42.8%, and 65.7%, respectively. While there was no healing with the lower beams until the surface temperature reached 100 °C. This is mainly related to the temperature gradient distribution. The asphalt binder in the lower beam had no ability to flow and heal the cracks at the lower surface temperatures of 60 °C and 80 °C. The temperature of the lower beam ranged from 32.4 °C to 47.0 °C when the surface temperature was 80 °C. As the temperature of the lower beams did not reach the softening point temperature of the binder, the cracks in the lower beams were almost not healed, and can be clearly seen after heating. The temperature of a small partial of the lower beam was higher than 47.5 °C when surface temperature reached 100 °C, so a low healing ratio of 12.6% was obtained in the lower beam. Based on the crack healing phenomenon and the corresponding healing ratios, it can be concluded that the gradient healing phenomenon of asphalt mixture exists with induction heating, and is mainly related to temperature gradient distribution within the sample. 

### 3.3. Heating Characteristics of Asphalt Mixture with Microwave Heating

**Figure 8:**
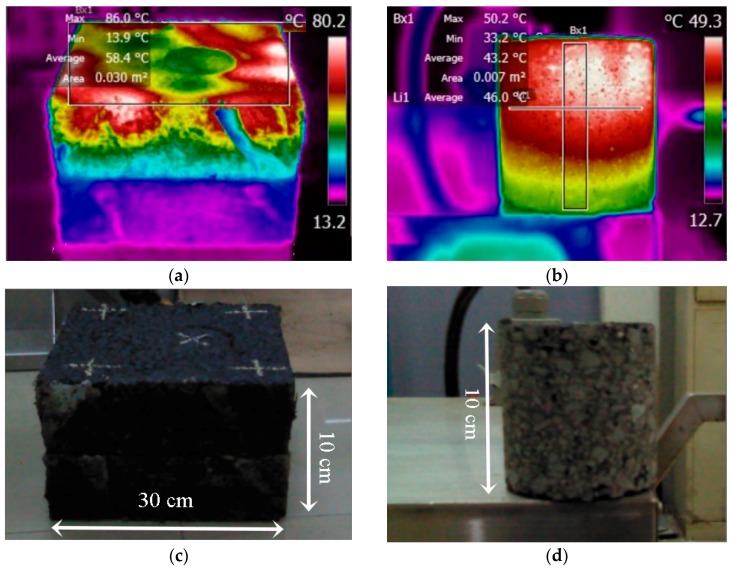
Infrared images of slab and core after heating for 15 min with improved microwave machine. (**a**) Surface temperature profile of slab after heating; (**b**) Vertical temperature profile of core after heating; (**c**) Slab sample; (**d**) Core sample.

Figure 8 shows the infrared image of the asphalt slab and core after heating for 15 min with the microwave machine. The average temperature of the slab surface reached 58.4 °C, and the temperature of the core ranged from 50.2 °C to 33.2 °C from top to bottom. In the top 7 cm of the core, the temperature ranged from 40.2 °C to 50.2 °C, with an average temperature of 45.4 °C, showing a much more uniform distribution than induction heating. The average temperature at the depth of 4 cm was 46 °C, around the softening point of the binder. It can be considered that the effective depth of the mixture was 4 cm after heating 15 min with the microwave machine.

After heating for 25 min, the average surface temperature of the slab was 91.2 °C, and the heating rate at the surface of the slab was 3.12 °C/min. The longitudinal temperature of the core ranged from 55.8 to 82.1 °C, which was higher than the softening point of the binder. This means that the heating depth has increased to the whole depth of the sample with a surface temperature lower than 100 °C. It can be concluded then that extending the heating time can increase the effective heating depth of asphalt mixture heated with the microwave equipment. 

To sum up, the heating functional range of the asphalt mixture with the microwave machine was more than 10 cm under 25 min of heating, which was much deeper than the maximum heating depth of induction heating (4.8 cm). However, the heating speed of the mixture under induction heating (17.5 °C/min) was much higher than that with microwave heating (3.12 °C/min). In addition, the uniformity of microwave heating was much higher than that of induction heating, which has a gradient distribution. The two heating methods have their own heating characteristics, which should be considered in practical application.

### 3.4. Induced Healing Performance of Asphalt Mixture with Microwave Heating

**Figure 9:**
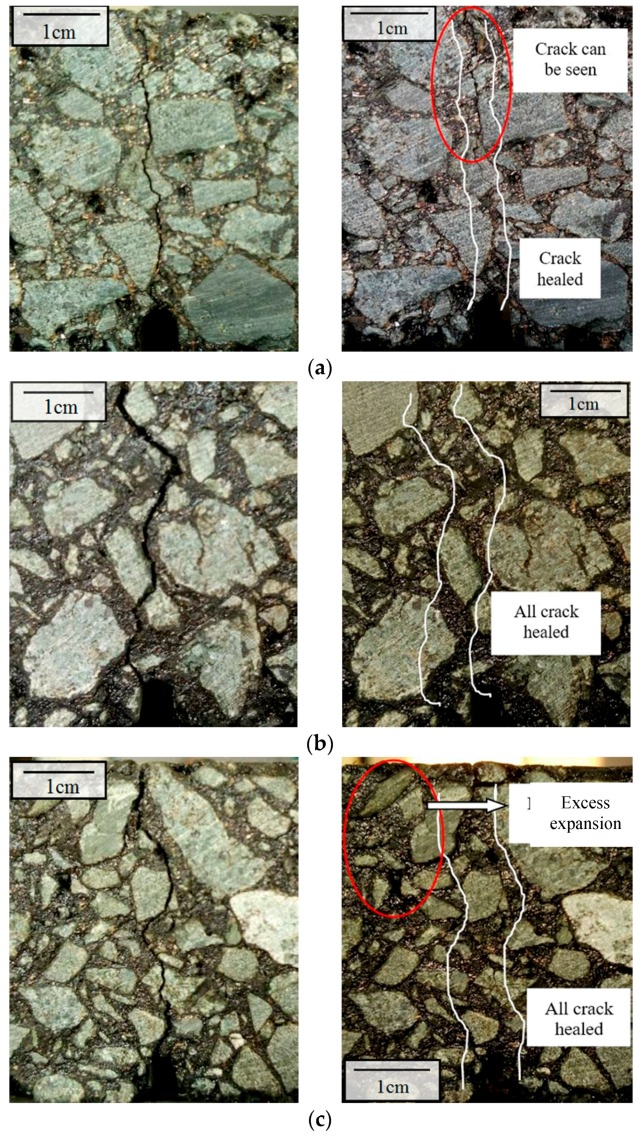
Healing phenomenon of asphalt concrete at different microwave heating temperatures. (**a**) Crack healing phenomenon at 60 °C: partially healed; (**b**) Crack haling phenomenon at 80 °C: fully healed; (**c**) Crack healing phenomenon at 100 °C: fully healed with excess expansion of the binder.

The cracks of the samples before and after microwave healing at different temperatures were shown in Figure 9. It can be seen from Figure 9 that the cracks are healed better with the increase of heating temperature. At a heating temperature of 60 °C, the crack was partially healed. When the heating temperature reached 80 °C, the crack was fully healed, showing a much better healing effect than that of induction heating. This difference can be explained according to the temperature distribution of the samples. The target heating temperature of the sample with induction heating was the surface temperature of the beams, and the beams showed a strong temperature gradient in the longitudinal direction. When the samples were heated uniformly with microwave heating, the temperature of the whole sample reached 80 °C, which is the reason why the crack can be healed all through the depth of the sample. It can be concluded that the healing phenomenon of the sample with microwave heating was much more uniform than that with induction heating. When the heating temperature reached 100 °C, distinct deformation of samples due to excess expansion of the mastic can be observed. For this reason, it is necessary to control the temperature during microwave heating. The deformation of the sample with induction heating only occurred in the surface of sample, due to the lower internal temperatures. While the whole sample heated with microwave heating suffered deformation because of the uniform heating. The deformation at the cracked areas will probably decrease the healing ratio.

The healing ratios of the samples at three different microwave heating temperatures are shown in Table 3. It can be seen from Table 3 that the upper and lower beams showed similar healing ratios and the healing ratio difference was less than 6%, indicating that the microwave-induced healing was quite uniform. The beams obtained their highest healing ratio at a heating temperature of 80 °C. A temperature of 60 °C was too low for asphalt binder to flow to heal all the cracks, while the healing ratio decreased at 100 °C due to excess deformation of the sample. It should be noted that some aggregates were broken during the bending test, which had a strong influence on the recovered fracture resistance of the beams. For this reason, the real crack healing efficiency of asphalt mastic should be much higher than the fracture resistance recovery ratio.

At the same heating temperatures, the samples heated with microwave heating showed quite different healing efficiencies from the samples heated with induction healing, due to the different temperature uniformities. The induction heating speed of the beams is much faster, but the heating depth is shallow, and there exists a strong temperature gradient, resulting in a poor or no healing effect in the lower beams. The microwave machine applies the radiation all around the samples, while the electromagnetic induction was applied only through the upper surface of samples. 

At the heating temperatures of 60 and 80 °C, the healing ratios of the lower beams were slightly higher than the upper beams. This can be attributed to the weight of the upper beams, because it has been reported that external loads have a positive effect on the healing of asphalt mixture [29]. The healing ratios of the beams decreased at 100 °C, especially the lower beam. In this case, the excessive thermal expansion of the asphalt mastic increased the internal pressure and damaged the structure. On the other hand, the weight of the upper beam contributed to the deformation of the lower beams, resulting in a decreased healing ratio. It can be concluded that the weight of the sample has a positive effect on healing at lower temperatures, but a negative effect on healing at excessively high temperatures.

## 4. Conclusions

The heating characteristics and induced healing efficiencies of asphalt mixture containing steel fibers under both induction and microwave heating were studied in this paper. Based on the results, the following conclusions can be drawn:

1. There is a strong temperature gradient through the thickness of asphalt sample heated with induction heating. The temperature difference between the surface and bottom of the sample increased with the heating time. An effective heating depth exists for each induction heating time, and the maximum effective heating depth of asphalt mixture with induction heating is around 48 mm, based on the allowable maximum surface temperature. It is inadvisable to increase the effective heating depth of the sample by extending the induction heating time, which will result in excess expansion deformation of the mixture.
2. Gradient healing phenomenon exists with the sample heated via induction heating, and is mainly related to temperature gradient distribution. The upper part of the sample healed much better than the lower part.
3. Microwave heating is relatively uniform in a longitudinal distribution, and the heating uniformity is independent of heating time.
4. Microwave heating causes more uniform crack healing in an asphalt mixture. The weight of the sample has a positive effect on healing at lower temperatures, but a negative effect on healing at excessively high temperatures.
5. Under the similar power and same radiation way, induction heating is fast and inhomogeneous in the longitudinal direction, while microwave heating is slow and uniform. The effective heating depth of microwave heating is much higher than that of induction heating.

## Figures and Tables

**Table 1:** The properties of asphalt binder and steel wool fibers.

Materials	Properties	Values	Specifications
Steel fiber	Length (mm)	4.2	-
	Equivalent diameter (μm)	70–130	-
	Gravity (g/cm^3^)	7.8	-
Bitumen	Penetration (25 °C, 100 g, 5 s, 0.1 mm)	68	60–80
	Ductility (15 °C, cm)	>100	100
	Softening point (°C)	47.5	47

**Table 2:** Induction healing ratios of the samples with different surface heating temperatures.

Beams	Surface Temperature		
	60 °C	80 °C	100 °C
Upper beam	30.8%	42.8%	65.7%
Lower beam	0	0	12.6%

**Table 3:** Healing ratios of the samples at different microwave heating temperatures.

Beams	Average Temperature		
	60 °C	80 °C	100 °C
Upper beam	30.3%	65.8%	62.9%
Lower beam	36.2%	70.9%	56.9%
